# The regional and global impact of the Russian invasion of Ukraine

**DOI:** 10.1016/j.lanepe.2022.100379

**Published:** 2022-04-01

**Authors:** The Lancet Regional Health – Europe

At the time of writing, there have been 2700 civilian casualties, including 1000 deaths (actual numbers are likely to be much higher), since the unprovoked Russian invasion of Ukraine on February 24, 2022. More than 3·6 million Ukrainian civilians have fled as refugees, mostly women and children (men aged 18–60 years are not permitted to leave the country). Cities, hospitals, and vital health infrastructure have been destroyed. As of March 22, WHO had verified a total of 64 attacks on health care and described it as an “act of unconscionable cruelty” and a violation of international humanitarian laws and human rights. “Every single attack on health care deprives people of life-saving services. The collapse of Ukrainian health system would be a catastrophe. Every effort must be made to prevent this from happening.”

The Ukrainian health system is under exceptional pressure, as demand has increased substantially due to a steep rise in casualties and humanitarian need. It is predicted that these challenges will be compounded in the days ahead, resulting from the destruction of water and sanitation infrastructure, inadequate vaccination coverage, overcrowding, and lack of access to medical care, all of which pose an imminent risk of the outbreak of infectious diseases such as measles, polio, tuberculosis, HIV, and diarrhoeal diseases. Additional disruption of medical supply chains and health services will result in increased mortality and excess illness due to non-communicable diseases such as cardiovascular disease, diabetes, and cancer. Maternal and neonatal morbidity and mortality will also increase due to lack of access to obstetric care—80 000 women are expected to give birth in Ukraine in the next 3 months. Services for mental health and psychosocial support are urgently needed to help people cope with the effects of the war. The war is also is also exacerbating the impact of the COVID-19 pandemic since only a third of the adult population in Ukraine is fully vaccinated.

The war has also destabilised the health care systems of neighbouring countries. The mass exodus of more than 3.4 million people from Ukraine is the fastest growing refugee crisis in the subcontinent. Neighbouring countries including Poland, Romania, Moldova, Hungary, Slovakia, Bulgaria, and the Czech Republic are particularly affected, with Poland being disproportionally impacted: more than 60% of all Ukrainian refugees have entered Poland in a short period of time, resulting in a monumental strain on its health-care system. These countries urgently need systemic solutions to move from spontaneous actions to the implementation of statutory measures to handle this crisis.

The United Nations High Commissioner for Refugees, WHO, UNICEF, United Nations Population Fund, and local governments are working relentlessly on the ground to ensure humanitarian aid and a constant flow of health supplies, however, the question is, how long can this continue? Data from the UN development programme projects that if the war continues, 90% of the Ukrainian population could be facing poverty and extreme economic vulnerability, “setting the country—and the region—back decades and leaving deep social and economic scars for generations to come.”

Although the war is in Ukraine and unprecedented economic sanctions are against Russia, the global economy, which has already been weakened by the COVID-19 pandemic, has also been negatively affected. Countries are witnessing severe disruption in the supply and rising prices of energy, raw materials, commodities, oil, food, and one of the worst disruptions to wheat supplies in a century. The war could have a devastating impact on hunger around the world, as Ukraine and Russia together account for a third of global wheat and barley exports and more than 70% of sunflower oil exports. Conflict-ridden countries and regions, including Lebanon, Yemen, Syria, Iraq, Afghanistan, north Africa, and west Africa, rely heavily on agricultural imports from Russia and Ukraine. The International Monetary Fund has said that this invasion could plunge the world's food supply into chaos, due to rising prices and the inability of Ukraine (where 70% of land is dedicated to agriculture)—the so-called breadbasket of Europe—to plant crops, including wheat.

As pointed out by David Leon and colleagues, these sanctions will make the economy of Russia and Belarus very isolated and unstable. Their health systems will face severe shortages, including shortages of modern therapies, many of which come from Western countries. Beyond this, the social, economic, and political stresses will worsen the living conditions of many people in Russia and Belarus and might potentially trigger increased mortality similar to that observed after the collapse of the Soviet Union in 1991.

The outcome of the war is far from certain. What is certain is that this war marks a turning point in the history that has disrupted the so-called long peace period since World War II with the possibility of returning to the era of war. Nuclear weapons, which have largely acted as a deterrent in preventing wars, have now become a conceivable reality. The course of modern day international geopolitics will change profoundly. The wave of insecurity among countries is leading to an increase in their defence budget and rightly so. The budget that could have otherwise gone towards health care, public health, education, and climate change will now fund weapons**.** What is also certain is that there will be no wins in this ‘single man’ inflicted disaster.

